# Recent Developments in Fabrication Methods and Measurement Schemes for Optically Pumped Magnetic Gradiometers: A Comprehensive Review

**DOI:** 10.3390/mi15010059

**Published:** 2023-12-27

**Authors:** Haifeng Dong, Hangfei Ye, Min Hu, Zongmin Ma

**Affiliations:** 1 School of Instrumentation and Optoelectronic Engineering, Beihang University, Beijing 100191, China; hfdong@buaa.edu.cn (H.D.); yehangfei1205@foxmail.com (H.Y.); humin_722@163.com (M.H.); 2 National Key Laboratory for Dynamic Measurement Technology and School of Semiconductor and Physics, North University of China, Taiyuan 030051, China

**Keywords:** magnetic field gradiometer, optically pumped magnetometer, intrinsic gradiometer

## Abstract

Optically pumped gradiometers have long been utilized in measurement in the International Geomagnetic Reference Field (IGRF). With advancements in technologies such as laser diodes and microfabrication, integrated gradiometers with compact sizes have become available, enabling improvements in magnetoencephalography and fetal magnetocardiography within shielded spaces. Moreover, there is a growing interest in the potential of achieving biomagnetic source detection without shielding. This review focuses on recent developments in optically pumped magnetic field gradiometers, including various fabrication methods and measurement schemes. The strengths and weaknesses of different types of optically pumped gradiometers are also analyzed.

## 1. Introduction

There are two primary motivations for measuring the distribution of magnetic fields. The first involves detecting magnetic field sources, such as in magnetocardiography (MCG), magnetoencephalography (MEG) and magnetic anomaly detectors (MADs). In these applications, gradiometer measurements with baselines ranging from millimeters to centimeters are commonly employed to minimize background common-mode fields. The second motivation puts more attention on the magnetic field image and/or spin polarization image itself, where spatial resolution can be down to microns or even nanometers [1,2,3,4,5,6,7,8]. In this review, we focus on the first case, i.e., a gradiometer based on an optically pumped vapor cell.

Magnetic field gradiometers are widely used in SQUID systems for biomagnetic imaging, both within magnetic shield rooms [9] and in unshielded environments [10]. Additionally, there have been reports on the development and utilization of magnetic field gradiometers based on pick-up coils [11] and TMR [12].

In this review, we primarily focus on magnetic gradiometers utilizing alkali atoms, which possess the capability to function in either a shielded near-zero field environment or an unshielded earth field environment. Early reviews of optically pumped magnetometers and their application in earth gradiometry can be found in references [13,14]; this reviews mainly include the development afterward.

**Figure 1:**
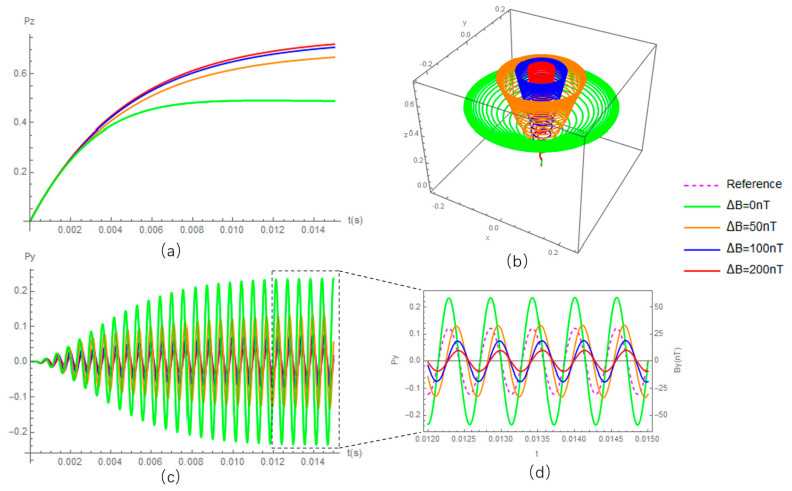
Precession of spin polarization under different main fields. (**a**,**c**) The transient solution of the spin polarization along the z-axis and y-axis, respectively. (**d**) A zoomed-in view of (**c**,**b**), showing the three-dimensional precession.

Although there are many kinds of OPMs, the working principle of all is based on the dynamics among the spin polarization vector, the oscillating field $B_{1}\cos\omega t$ (along the y-axis in Figure 1), the main field and light pumping (along the z-axis in Figure 1) and relaxation. Figure 1 illustrates the precession of spins under the different main fields of $B=B_{0}-\Delta B$, where $B_{0}=\omega \gamma$ and γ are the gyroscope ratio of the particle. From Figure 1a, it is clearly shown that the projection of spin on the z-axis (Pz) changes with the main field, which provides one mechanism for detecting B, i.e., the so-called Mz magnetometer. One not-so-obvious phenomenon in Figure 1 is the phase difference between spin precession and reference oscillating field $B_{1}\cos\omega t$, which gives another measurement mechanism of the main field, i.e., the so-called Mx magnetometer. Similarly, $B_{1}$ can also be measured if the main field, B, is kept constant: i.e., the radio-frequency magnetometer called the Hanle effect magnetometer is used in special cases when the main field is equal to zero.

The measurement of the field gradient can be achieved through a simple approach involving two magnetometers with an appropriate baseline, the optimal value of which has been discussed in [15]. Other techniques, such as the feedback method and the intrinsic method, have also been proposed. The subsequent sections of this review classify optically pumped gradiometers based on their background fields and the differential methods employed.

## 2. Optically Pumped Gradiometer under Near-Zero Field

For measuring in near-zero field conditions, the spin exchange relaxation-free (SERF) regime is preferred due to its ability to enhance signal strength and reduce spin-projection noise by providing a long transverse relaxation time [16]. The first highly sensitive optically pumped magnetometer (OPM) operating in the SERF regime was demonstrated using two orthogonal light beams. One beam was used to pump the spins, while the other was used to probe the spin polarization along the probe beam [17]. The detected vector field was perpendicular to the plane defined by the pump and probe beams. This scheme also achieved a benchmark sensitivity, of 0.16 fT/Hz^1/2^, for OPMs [18].

The use of a single light beam with an orthogonal modulation field is a prevalent choice for small-sized magnetometers due to several advantages. Firstly, employing a single light beam enables smaller dimensions than the two-orthogonal-beam scheme and facilitates the assembly process. Additionally, this scheme involves modulating signals with high frequency, typically ranging from a few to several decades of kilo-hertz. This high-frequency modulation helps mitigate the impact of 1/f noise and enhances the signal-to-noise ratio [19].

A basic sensing model for the dual-orthogonal-beam scheme and the single-beam scheme can be deduced with the Bloch equation. In the first case, the frequency response of polarization along the x-axis can be expressed as follows [20]:

(1) $$P_{x}\left(\omega \right)=P_{0}\gamma \frac{\Delta \omega }{\omega^{2}+\Delta \omega^{2}}B_{y}$$

where $P_{0}=R_{p}/(R_{p}+R_{2})$, *R*_p_ and *R*_2_ are the pumping rate and transverse relaxation rate, respectively; γ is the gyromagnetic ratio; ω is the frequency of the signal; and $\Delta \omega =R_{p}+R_{2}$ is the half-width at half maximum.

For the single-beam orthogonal modulation scheme, a modulation field vertical to the light beam is usually added; thus, the dynamic solution for $P_{Z}(\omega )$ is [21,22]

(2) $$P_{Z}(\omega )=-\frac{2P_{0}\gamma }{\Delta \omega }J_{1}J_{0}B_{X}\left(\omega \right)\sin\omega_{m}t$$

where $J_{1}$ and $J_{0}$ are the Bessel functions with a variable equal to the ratio between modulation amplitude $B_{1}$ plus gyromagnetic ratio γ and the modulation frequency $\omega_{m}$.

From Equations (1) and (2), we can see that the frequency response is limited by the linewidth, Δω, and the modulate frequency, $\omega_{m}$, respectively.

### 2.1. Gradiometer Composed of Separate Magnetometers

**Figure 2:**
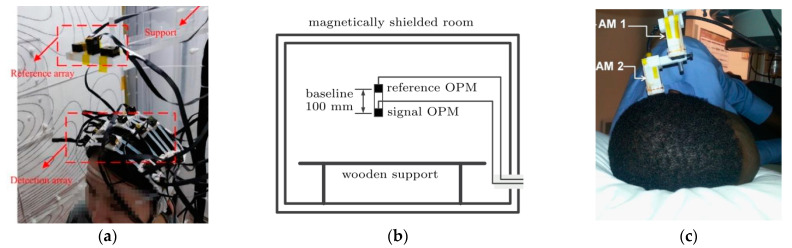
OPM gradiometer system using Qu-spin magnetometers for (**a**,**c**) MEG [24,32] and (**b**) MCG [25].

Even with high sensitivity under the SERF regime, gradiometers are preferable to eliminate unexpected environmental magnetic-field noise interferences in many applications, such as in MEG-based decoding analysis [23]. The direct way to realize a gradiometer is to use separate magnetometers with an approximate baseline from the signal source to map the environmental interference. As shown in Figure 2a, three Qu-spin magnetometers were used to measure $B_{x}$, $B_{y}$ and $B_{z}$ of the environmental magnetic field noise [24]. Figure 2b is a similar system used for MCG [25]. According to reports, an MCG signal can be sensed by the reference OPM even with a 100 mm baseline [25], and an MEG signal can be observed even the sensor is 20 mm away from the scalp [26]. The least squares subtraction method was used in references [24,25], while other approaches in the SQUID-based system may be constructive once the sensor number is more than two [27,28]. Reports of optically pumped magnetometer arrays, which may be thought of as gradiometers in planes or curved surfaces, can be found in references [29,30,31].

A gradiometer composed of separate magnetometers will obtain a magnetic field gradient through the subtraction of voltage signals. The ratio of the voltage signal to the magnetic field signal will be the scale factor, and the mismatch in scale factors will reduce the common-mode rejection ratio (CMRR). The scale factor here can be characterized using the least squares method once all data are recorded. To enable real-time detection, a feedback method has been proposed.

### 2.2. Gradiometer Using Feedback Method

The feedback method presents an alternative means of detecting magnetic field gradients. This approach differentiates the magnetic field signal directly, eliminating the need to employ the least squares method to minimize scale factor differences. By utilizing a reference sensor to measure the background field and counteracting it with compensation coils, this method enables the direct output of the gradient magnetic field via the second magnetometer.

**Figure 3:**
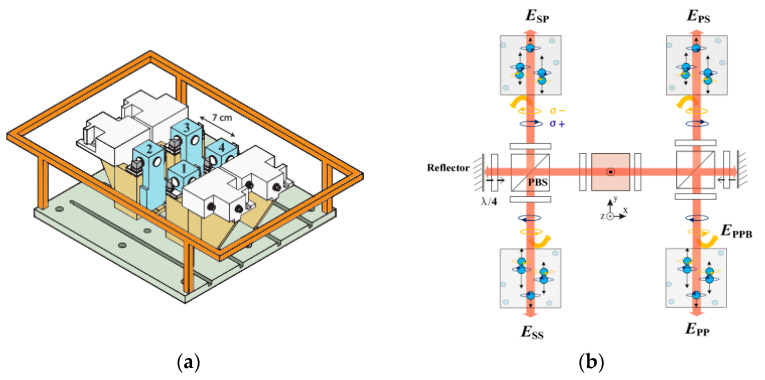
(**a**) Scheme of a SERF gradiometer where four magnetometers are symmetrically located in the plane of a single feedback field coil [33]; (**b**) scheme of a four-channel gradiometer using parametric modulation measurement [35].

Robert Wyllie et al. used this method and a self-made sensing head to measure fetal magnetocardiography [33]. Figure 3a shows the gradiometer scheme of four sensing heads with a baseline of 7 cm, one of which is used for feedback to the plane coil. Large coils (with a length of 3 m each) are used to actively cancel the residual field. In this way, the Fetal Magnetocardiography (FMCG) signal is detected in real time. I.A. Sulai et al. demonstrated another, similar scheme later, with a baseline of 4 cm in a magnetically shielded room (MSR), for fetal magnetocardiography using two vapor cells and orthogonal beams [34]. Ziqi Yuan has designed a compact four-channel, single-beam, parametric-modulated, optically pumped gradiometer with a baseline of 1.5 cm, the scheme of which is shown in Figure 3b [35]. Joonas Iivanainen et al. have proposed an array of optically pumped magnetometers for magnetoencephalography, with static and dynamic noise signals measured with reference sensors and fed back to an active three-axis magnetic compensation system [36].

**Figure 4:**
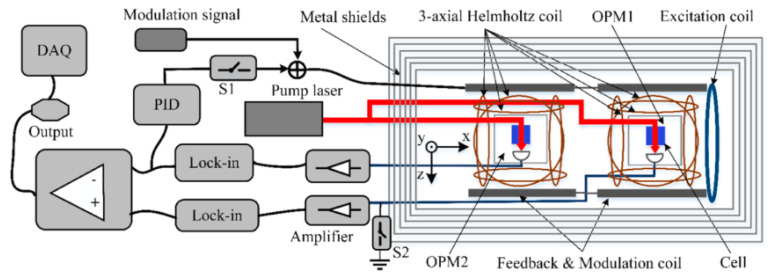
Feedback method using single-beam perpendicular modulation to detect a field [37].

Besides the real-time problem of using the least squares method with separate magnetometers, Yang Wang et al. pointed out that the strong common-mode magnetic field limits the gain of amplifiers in the signal condition circuit and wastes most of the effective digits of the analog-to-digital (ADC) converter [37]. Closed-loop compensation can solve this problem. Figure 4 shows the closed-loop compensation scheme using parametric modulation to detect the field where the final differentiation seems to be unnecessary. This idea is similar to that in reference [33], i.e., using the output of one vapor cell to compensate for the field around both vapor cells. The result would be that the field around one vapor cell would be close to zero and the field around the other would be equal to the gradient, which would be measured directly. In this way, the measurement range of the magnetic field gradiometer could be very small.

If a self-made gradiometer is used instead of a commercial magnetometer and there are coils controlling the field around the cell and/or cells, one can easily select between the least squares method and the feedback method.

**Figure 5:**
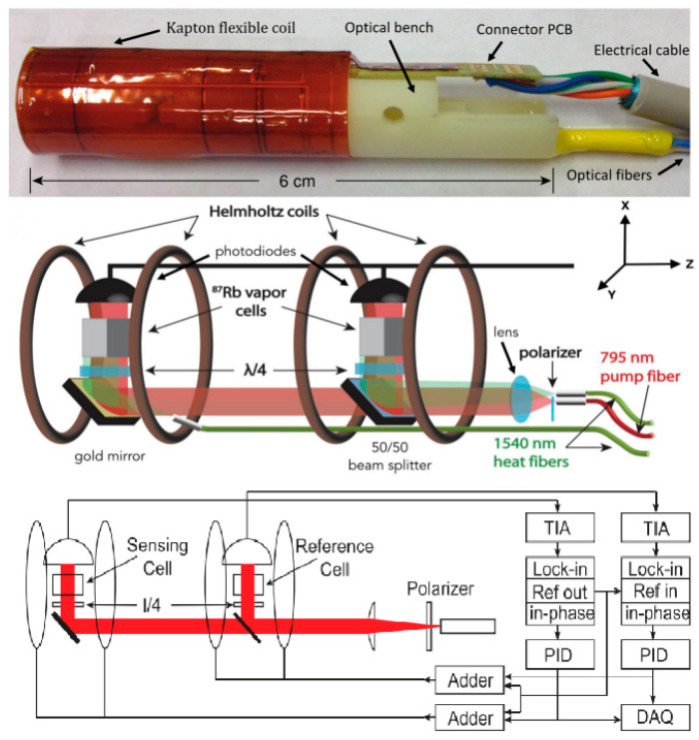
Magnetic field gradiometer photo, the inside structure with a baseline of 2 cm and the electrical signal processes [38,39].

In 2017, D. Sheng et al. designed an atomic magnetic gradiometer using two microfabricated vapor cells, vacuum thermal isolation and laser heating [38]. To shrink the total size of the sensing head and increase the common noise rejection ratio, one light source was split into two beams with a dichroic mirror to pump and probe the spin in the two vapor cells, as shown in Figure 5. Another two 1550 nm laser beams were used to heat the cells separately. The dichroic mirror was also used as the reflector of the 1550 nm light.

A method of measuring common-mode noise rejection ratios is discussed in reference [38]. By putting the gradiometer in a shield environment and adding a large enough Helmholtz coil system, the field in the uniform area generated by the coils could be thought as a perfect common-mode signal. Then, white noise with a low pass filter would be inputted to the coil. The ratio between the output of the single channel and that of the differential channel would be characterized as the CMRR. It is worth noting that during this characterization, the input field should be kept the same for both channels and the gradiometer output noise should be higher than the environment gradient noise. Otherwise, the measured CMRR may be smaller than its real value.

Here, we want to point out that there is a problem with this feedback method, i.e., there is always a practical net difference, especially for high dynamic responses. Considering that the gradient would be much smaller than the common field in the earth environment, the common field variation could cause a significant contribution to the gradiometer’s noise, making the CMRR become frequency-dependent.

### 2.3. Intrinsic Vector Gradiometer

Intrinsic differentiation is another way to solve the problem of the non-real-time gain limitation of the amplifier and the waste of the effective digits of the ADC. There will be no net difference problem with the feedback method, and no compensation coils will be needed.

The gradient measurement of the intrinsic vector gradiometer is founded on the differential of optical signals. In 2015, Keigo Kamada et al. proposed an intrinsic near-zero field gradiometer that differentiates optical rotation angles by reflecting twice before the probe beam re-enters the cell in a different position [40]. The Jones matrix of a reflector mirror with a coordinate system defined by PS vectors is

$${\left[\begin{array}{cc} 1 & 0\\ 0 & e^{-i\pi } \end{array}\right]}_{.}$$

**Figure 6:**
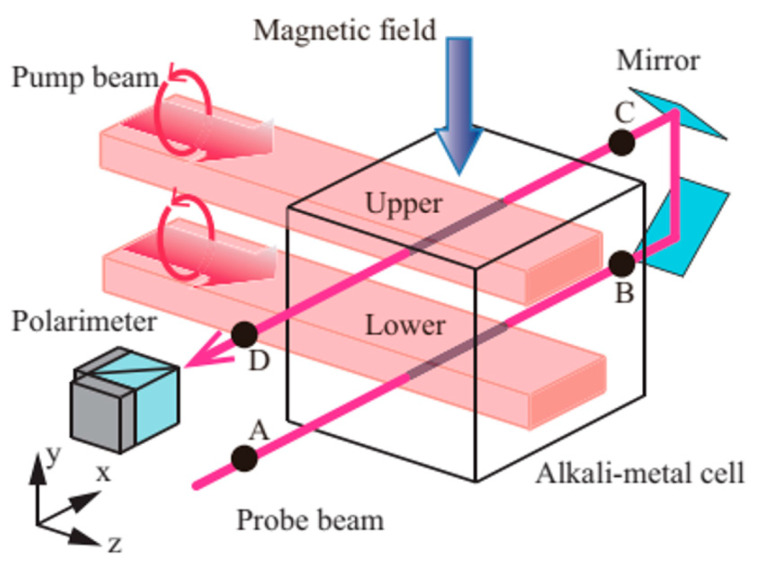
Optical scheme of an orthogonal beam intrinsic gradiometer [40].

Thus, the Jones matrix of two reflector mirrors is

$$\left[\begin{array}{cc} 1 & 0\\ 0 & 1 \end{array}\right]$$

which means that the optical rotation angle is kept the same after two reflections. As the light propagation direction is opposite, the optical rotation angle for the second position is also opposite to the previous position in the earth coordinate system. In this way, the optical rotation angles in two positions are differentiated directly. Figure 6 shows the optical paths of the gradiometer. Other kinds of intrinsic OPM gradiometer that work with the earth field will be discussed in the next section.

However, as the scale factor, namely the conversion factor from magnetic to optical signals, varies between different vapor cells or different positions within the same vapor cell, the obtained optical signal will contain common-mode magnetic-field noise proportional to the difference of the scale factors. The variance in the scale factors is inherently linked to the unique characteristics of the vapor cells and cannot be rectified through the least squares method or translated into a field difference via the feedback method.

### 2.4. Gradiometer with a Short Baseline under a Near-Zero Field

**Figure 7:**
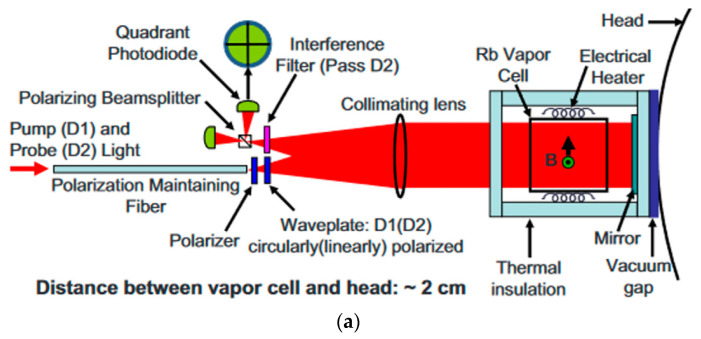

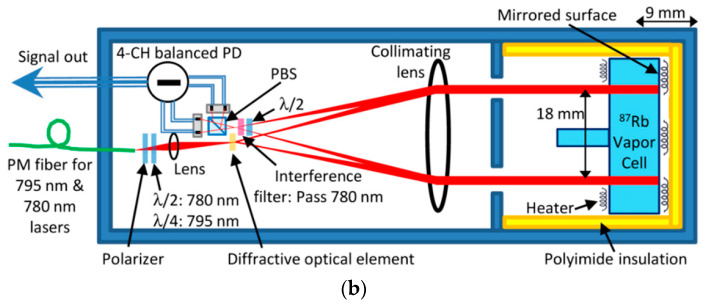
(**a**) Schematic of a gradiometer using a two-color pump/probe scheme [43]; (**b**) schematic of a gradiometer using a diffractive optical element and one vapor cell [44].

Most of the gradiometers discussed above use two vapor cells each. Using only one vapor cell has the benefit that the alkali atom density and buffer gas pressure will be kept the same all the time [41,42]. Furthermore, with a small baseline, it is easier to calibrate the noise floor of the gradiometer. In 2010, Cort Johnson et al. of Sandia Labs implemented a compact gradiometer using a two-color pump/probe scheme where pump/probe detuning and power could be independently adjusted to optimize performance [43]. A four-quadrant-channel diode array was used to separate the sensing area. By differentiating the signals from different quadrant channels, the magnetic field gradient could be detected. With the effective suppression of common-mode noise, a sensitivity of less than 5 fT/Hz^1/2^ was achieved. The schematic of the sensor is shown in Figure 7a. The baseline was limited by the cross-section of the pump–probe beams. In 2016, Anthony P. Colombo et al. of the same lab increased the baseline by using a diffractive optical element to separate the pump–probe light into four first-order beams. In this way, they obtained a baseline of 1.8 cm in a single vapor cell [44]. The schematic of the optical path is shown in Figure 7b. Using a microfabricated process, Austin R. Parrish et al. designed a gradiometer in which microchannels connect two blown-glass vapor cells with a baseline of 0.55 cm [45].

## 3. Optically Pumped Gradiometer under Near-Earth-Field Conditions

For measuring in near-earth-field conditions, this gradiometer has extensive applications, such as an MAD of the earth’s surface due to crustal field changes, core changes, extreme and ultra-low-frequency magnetospheric disturbances and surface electromagnetic effects associated with earthquakes and volcanic activity [46]. Recent research has shown its ability to detect biomagnetic field sources in unshielded environments.

While most zero-field optically pumped magnetometers detect vector fields defined by light and controlled field directions, gradiometers that work in near-earth-field environments can be both scalar and vector. It is easier to set up a scalar gradiometer, while a three-axis vector gradiometer can obtain more information about the magnetic field source.

Working with a three-layer magnetic shield, Dong Sheng et al. characterized the sensitivity of the microfabricated gradiometer under a bias field from 2500 nT to 15,000 nT after zeroing the bias field using the sensor’s compensation coils. According to the quadratic scaling law of gradiometer noise with the bias field, the sensitivity of the gradiometer was expected to be around 0.5 pT/cm/Hz^1/2^ at the earth field amplitude [38].

**Figure 8:**
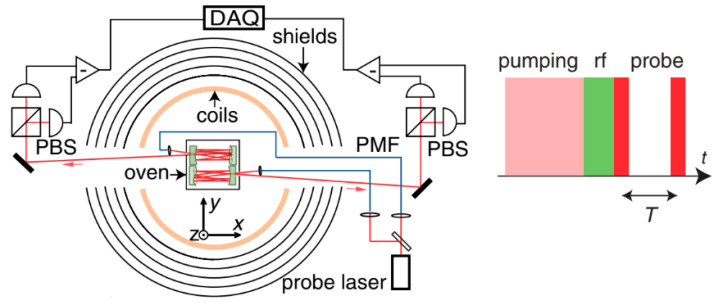
Gradiometer using two multipass cells (**left**) and the measurement time sequential (**right**) [47].

Back in 2013, Dong Sheng et al proposed an OPM gradiometer using two multipass vapor cells with a baseline of 1.5 cm and a dark measurement of the Larmor frequency, which enabled a sensitivity of 0.54 fT/Hz^1/2^: a record for the scalar magnetometer. Although it was characterized in the shield, there was a bias magnetic field of 7290 nT. Figure 8 shows the optical path of the scheme, the time sequence for pumping and the rf tipping signal and the probe signal, respectively [47].

### 3.1. Gradiometer in the Earth Environment

In 2010, Polatomic inc. took charge of a SBIR/STTR project named the Laser Femto-Tesla Magnetic Gradiometer with the objective to explore temporal variations and gradients in the magnetic field at the earth’s surface. The designed dynamic range was from 25,000 nT to 75,000 nT [46,48].

**Figure 9:**
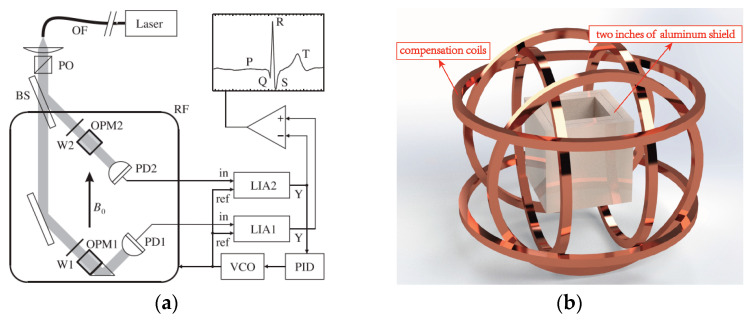
(**a**) Scalar gradiometer scheme in a partially shielded room [49] and (**b**) the structural schematic of the two-inch-thick aluminum shield and the compensation coils used in SERF magnetometer.

The major challenge for optically pumped gradiometers working in earth environments is oscillating and randomly fluctuating ambient fields. A gradiometer is a kind of active method to suppress environmental signals. Partial shields are also used when the CMRR is not high enough to detect the magnetic source, such as a cardiomagnetic field. Figure 9a shows a partially shielded Mx scalar gradiometer with a single 1-millimeter-thick layer of mu metal and 8-millimeter-thick copper-coated pure aluminum, which suppresses 50 Hz components by a factor of 150. Magnetocardiography is recorded with the this passive shield and active gradient measures [49]. The first demonstration of a SERF magnetometer working in an earth environment used a 2-inch-thick aluminum shield, as shown in Figure 9b [20,50]. Carolyn O’Dwyer proposed a feed-forward method to suppress the 50 Hz-line periodic noise when feedback is invalid due to the limited frequency response [51]. Considering that 50 Hz is a single-frequency signal, there may be other ways to compensate for this noise, such as designing a special Kalman filter.

**Figure 10:**
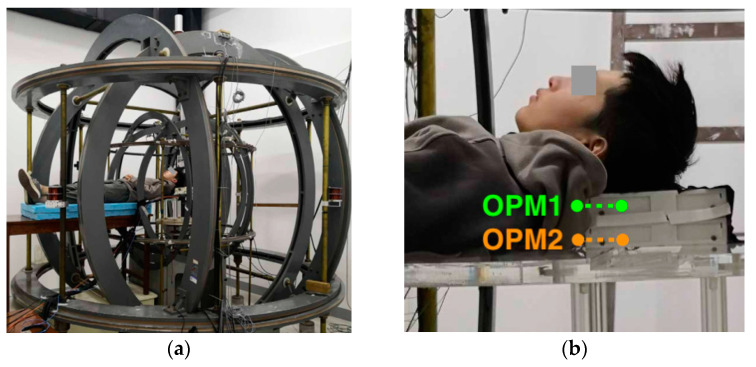
(**a**) Large coils for MEG in an earth environment and (**b**) a local enlarged image showing the positions of OPM1 and OPM2 [52].

In 2020, two kinds of OPM gradiometers that could measure the MEG in earth environments were realized. Rui Zhang et al. of Peking University demonstrated a MEG gradiometer in the earth’s field environment, where the horizontal components of the earth’s field were actively shielded by large coils with a diameter of several meters. A Bell–Bloom amplitude-modulated non-linear magneto-optical rotation (NMOR) method was used to measure the remaining vertical field. The baseline of the two sensors was 6 cm. The large coils and the sensor positions are shown in Figure 10a,b, respectively. Those authors used the differential method and the feedback method to obtain the field gradients [52].

**Figure 11:**
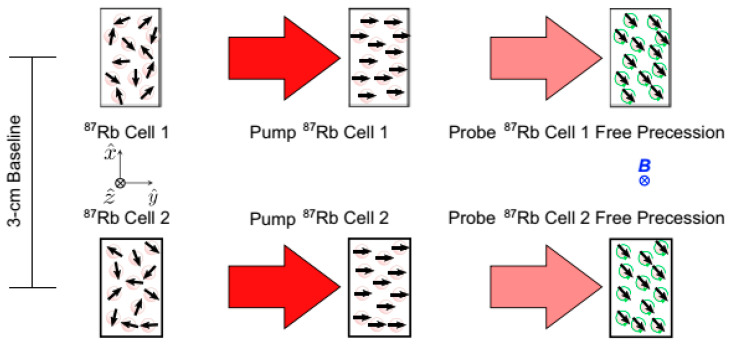
The scheme of pulse pumping/free decay scalar gradiometer [53].

Another scheme was proposed by M.E. Limes et al. from Princeton university. Those authors measured the MEG and MCG signals in an earth magnetic field environment without using active magnetic shielding [53]. Two cells were used, with a baseline of 3 cm. A short, high-power pulse was used to pump the Rb to spin at close to one hundred percent [54], and the FID signal was measured using a far-detuned probe beam. The pulse pumping beam and the probe beam were on the same axis, which was the only dead axis. The three stages, initial, pumping and free precession, are illustrated in Figure 11. The first-order magnetic field gradient signal was obtained by subtracting the frequencies recorded from two alkali vapor cells and dividing the result by the 87Rb gyromagnetic ratio. Richard J Clancy et al. has discussed how well a dipolar source could theoretically be localized through field gradient detection without shielding [55].

**Figure 12:**
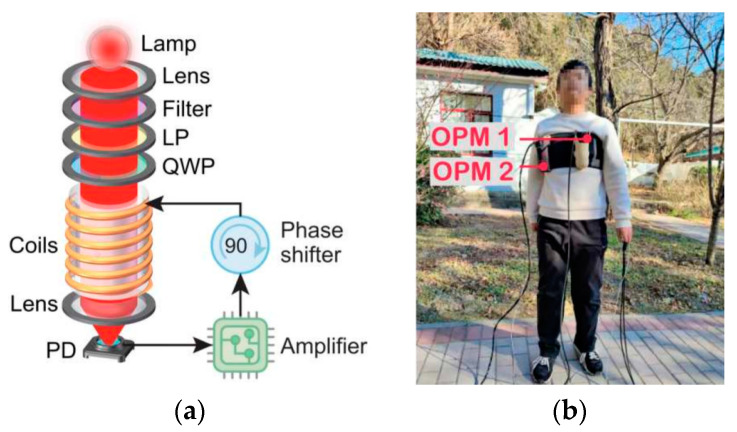
(**a**) The self-oscillating sensing scheme of the OPMs and (**b**) their assembly positions on the chest [56].

In 2023, Wei Xiao et al. proposed a movable gradiometer based on a self-oscillating sensing mechanism that would work in an earth field environment [56]. MCG signals were detected during a subject’s high-knee exercise. Figure 12 shows the basic scheme of the OPMs and their positions during that test.

In 2013, Shuhe Wu et al. proposed a magnetic field gradiometer using quantum-enhanced technology. That gradiometer would have eliminated ambient magnetic noise and photon shot noise at the same time. Two beams of light were quantum-mechanically entangled through four-wave mixing (FWM) to squeeze intensity-difference noise [57,58]. The possibility of realizing subshot-noise magnetometry in practical applications was demonstrated.

The scalar gradiometer presents a natural advantage in terms of its equal scale factors of two cells, i.e., the gyromagnetic ratio of alkali atoms. However, insensitivity to vector gradients and tensor information have hindered further enhancements in magnetic source detection. In reference [52], a vertical field was measured through compensation of the horizontal field, while the transverse field could not be detected simultaneously.

### 3.2. Intrinsic Scalar Gradiometer

Compared to external gradiometers, which use two separate magnetometers, intrinsic gradiometers differentiate signals before they are converted to voltage. When working in an earth-scale magnetic field, an intrinsic gradiometer will become more important because the ratio between the background field and the gradient will be larger than that of the gradiometer in a zero-field environment.

**Figure 13:**
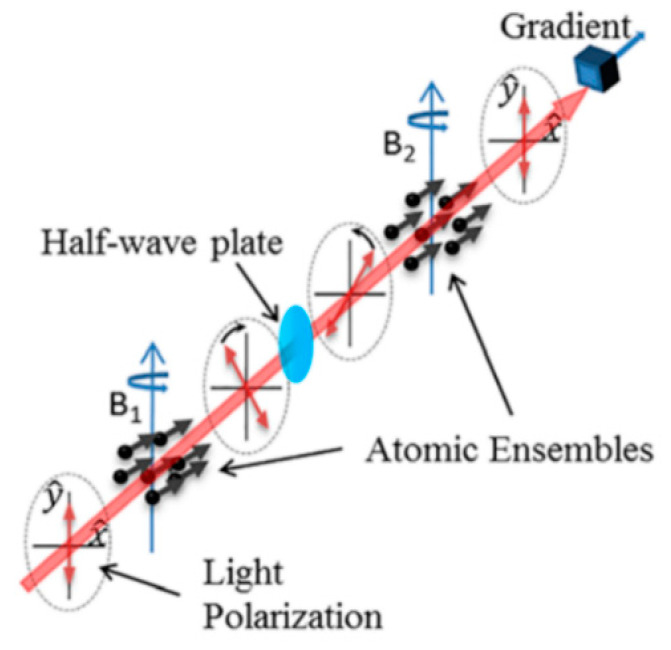
The probe path of an intrinsic, scalar, optically pumped gradiometer using a half-wave plate to reverse optical rotation [59].

Rui Zhang of Geometrics Inc. discussed a kind of intrinsic, scalar, optically pumped gradiometer using a half-wave plate to reverse the rotation angle of the polarization plate of a probe beam [59]. Figure 13 shows the probing scheme, where the rotation angle of the polarization plate of the probe beam after two cells is

(3) $$\begin{array}{l} \theta =k\left[-P_{1}\sin\left(\omega t+\varphi_{1}\right)+P_{2}\sin\left(\omega t+\varphi_{2}\right)\right]\\ \;\approx k\frac{P_{1}+P_{2}}{2}\left(\varphi_{2}-\varphi_{1}\right)\cos\left(\omega t+\frac{\varphi_{1}+\varphi_{2}}{2}\right) \end{array}$$

where *k* is the proportional coefficient; *P*_1_ and *P*_2_ are the spin polarizations of cell 1 and cell 2, respectively, which are approximately equal in resonance; and $\varphi_{1}$ and $\varphi_{2}$ are the respective phase differences of the spin polarization for the modulation signal. The magnetic field gradient can be measured directly from the out-of-phase demodulation signal of the polarization rotation of cell 2.

The gradient signal can feed back to control the modulation frequency difference between two cells or the local field of either cell. It is worth noting that if one cell is used to control the common parameter, such as the modulate frequency or field covering both cells, then the gradiometer will turn into a feedback type and the half-plate will become irrelevant. Using this half-wave-plate intrinsic differential method, Robert J. Cooper et al. later designed an intrinsic radio-frequency gradiometer [60].

**Figure 14:**
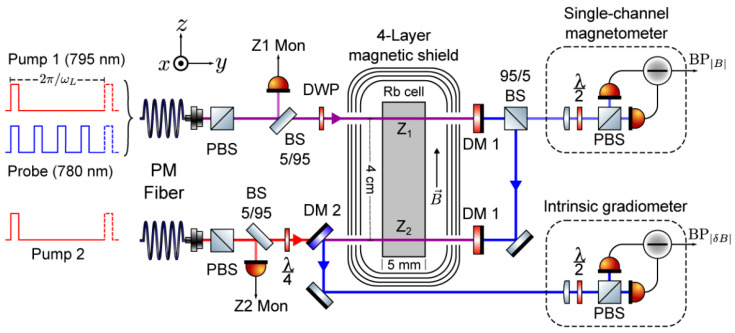
Optical scheme of the intrinsic scalar gradiometer with two mirror reflections [61].

A.R. Perry proposed another kind of intrinsic, optically pumped gradiometer, which would use the two-mirror-reflection method discussed in reference [40]. That gradiometer was based on amplitude-modulated Bell–Bloom pumping so that it could measure the scalar field [61]. It was tested in a shield with a bias field of 22,000 nT and a baseline of 4 cm in a single vapor cell. Figure 14 shows the optical scheme of the gradiometer. The output signal can also be analyzed using Equation (3). The probe can work in pulsed mode. Demodulation is achieved by directly differencing the signal over the π/2 and 3π/2 phases. The demodulation signal is proportional to the value of the phase difference between $\varphi_{2}$ and $\varphi_{1}$ in Equation (3).

**Figure 15:**
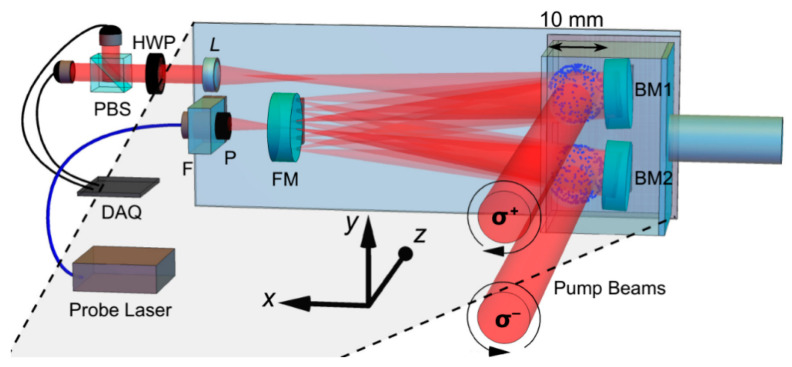
Scheme of the opposite-pumping V-shaped multipass gradiometer [62].

The third kind of intrinsic gradiometer is based on opposite pumping with right-circularly and left-circularly polarized light [62]. In this way, the phase of the precession of two regions will have a difference of π, leading to the same gradient of optical rotation as with Equation (3) in the Bell–Bloom mechanism. An extra decay term should be added to Equation (3), and the precession frequency will be different for the two terms in the free-decay mechanism. The measurement would be performed in the magnetic shield under a bias field of 26,000 nT and a baseline of 1.4 cm in a single vapor cell. Figure 15 shows the optical path of the gradiometer, where the V-shaped multipass design of the probe beam is realized to increase the optical depth. In 2023, S.Q. Liu et al. compared the intrinsic gradiometer based on the half-plate method and the opposite pumping method and measured the field gradient tensor using the modulated field and scalar output [63,64,65].

### 3.3. Gradiometer with a Short Baseline in a Near-Earth-Field

**Figure 16:**
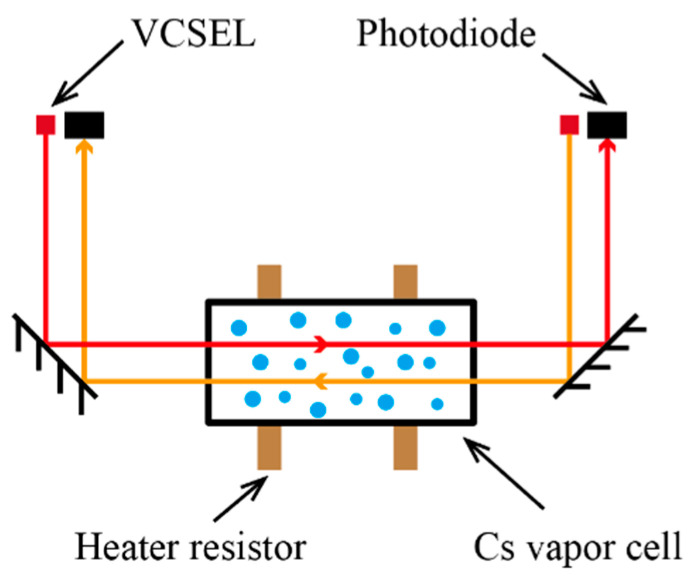
The optical path of the frequency-modulated Bell–Bloom OPM.

Although a certain baseline is necessary for magnetic field source detection [66], a short baseline is preferred to test the instrumental noise floor. Rui Zhang et al. of Geometrics Inc. demonstrated a gradiometer using two miniature scalar OPMs with a size of 2.5 cm × 2.5 cm × 3 cm each. The optical path of the OPM is shown in Figure 16; the pumping light worked in a frequency-modulated Bell–Bloom mode to eliminate crosstalk. The baseline was 2.5 cm when the two OPMs were placed next to each other. A sensitivity of 1 pT from 2 Hz to 50 Hz was achieved in a typical commercial environment without any mu-metal or aluminum shielding [67].

**Figure 17:**
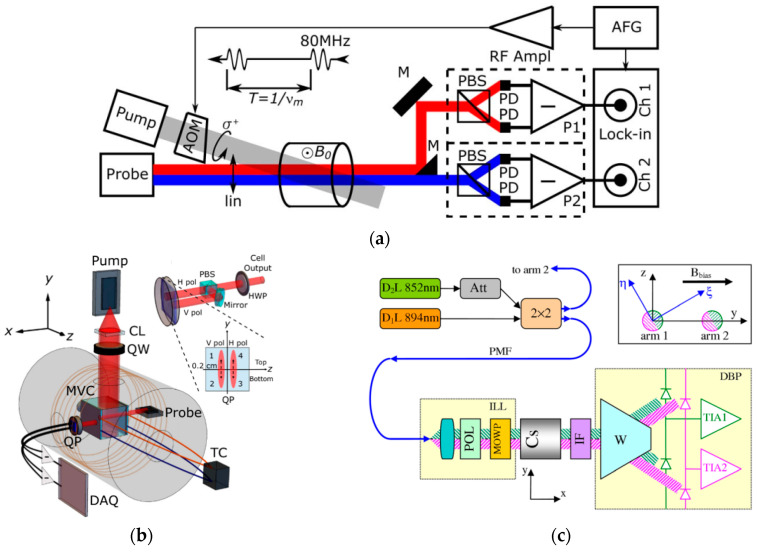
Short-baseline gradiometer detecting different parts of the probe beam: (**a**) amplitude-modulated pumping and near coincidence of the pumping and probe beam [69]; (**b**) optical path of the pulsed pumping and free decay gradiometer [68]; and (**c**) frequency-modulated pumping and completely coinciding scheme realized with a coupler, a multiorder wave plate and an interference filter [70].

To achieve a short baseline down to 0.5 cm, some researchers detected different parts of the probe beam directly [68,69,70], as shown in Figure 17. Using pulsed pumping, a free decay scheme and a baseline of 0.2 cm, V.G. Lucivero achieved an OPM gradiometer with a common-mode rejection ratio of higher than 10^4^ and nearly quantum-noise-limited sensitivity in earth-scale fields [68]. Yu Ji et al. used adjacent microfabricated RF coils to measure the magnetic fields of different positions sequentially, then obtained the gradient with a short baseline [71].

## 4. Conclusions and Perspective

In magnetic-field shielding space, optically pumped gradiometers can effectively reduce background noise. The differential signals can be electric, photonic or magnetic. In the first case, separate magnetometers are used with an appropriate baseline. In the second case, an intrinsic-gradiometer method involves differentiating the optical rotations of two vapor cells directly before converting them to voltage. In the case of magnetic differentiation, a reference OPM is used to compensate for the common environment field, allowing the direct detection of field gradients from another OPM.

Under near-earth field conditions, optically pumped gradiometers can measure precession Larmor frequency through various methods, such as free decay, self-oscillation, Bell–Bloom and Mx. These systems have the advantage of maintaining the same scale factor for two OPMs, i.e., the gyromagnetic ratio. Nonetheless, intrinsic scalar gradiometers remain attractive due to their direct output, possible higher signal amplifying gain and lower waste of bits during analog-to-digital conversion. For an intrinsic scalar gradiometer, the ratio between field detuning and the phase difference (between stimulation and spin precession signals) should be kept the same for the two cells; otherwise, there will be bias output for the same field detuning.

Although extensive research has been conducted on optically pumped field gradiometers, as we have outlined above, several unresolved issues must be addressed to facilitate wider applications. For direct magnetic-field differentiation using the feedback method, the net difference, especially in the high frequency range, will impede the enhancement of the CMRR and, consequently, the sensitivity of the gradiometer. On the other hand, the precise consistency of scale factors is essential when differentiating photonic and electronic signals. While the scale factor of scalar measurement relies on the gyromagnetic ratio, a fundamental physical constant, the locked frequency may change due to a light shift and/or phase shift. A promising approach to achieving an exceptionally sensitive scalar gradiometer involves adopting short-pulse pumping and a free induction-decay measurement scheme. However, the current lack of commercial maturity in high-power, short-pulse pumping diodes presents a significant obstacle. Furthermore, two critical areas necessitate increased focus: making technical noise in the common mode and establishing diverse scales for the common mode and differential signals. The successful resolution of these issues, combined with advancements in key devices and fabrication methods, will result in optically pumped field gradiometers finding widespread applications in clinical diagnostics, including magnetocardiography and magnetoencephalography with or without magnetic shielding, as well as other magnetic anomaly detections.

## Data Availability

Data are contained within the article.
